# The protective effect of amifostine on ultraviolet B-exposed xeroderma pigmentosum mice

**DOI:** 10.3332/ecancer.2010.176

**Published:** 2010-09-22

**Authors:** SL Henry, D Christiansen, FR Kazmier, CL Besch-Williford, MJ Concannon

**Affiliations:** 1 Division of Plastic Surgery, University of Missouri, One Hospital Dr, Columbia, MO, USA; 2 Veterinary Pathobiology, University of Missouri, One Hospital Dr, Columbia, MO, USA

## Abstract

**Background:**

Amifostine is a pharmaceutical agent that is used clinically to counteract the side-effects of chemotherapy and radiotherapy. It acts as a free radical scavenger that protects against harmful DNA cross-linking. The purpose of this study was to determine the effect of amifostine on the development of skin cancer in xeroderma pigmentosum (XP) mice exposed to ultraviolet B radiation (UVB).

**Methods:**

Twenty-five XP mice were equally divided into five groups. Group 1 (control) received no amifostine and no UVB exposure. Group 2 also received no amifostine, but was exposed to UVB at a dose of 200 mJ/cm^2^ every other day. The remaining groups were subjected to the same irradiation, but were given amifostine at a dose of 50 mg/kg (group 3), 100 mg/kg (group 4), or 200 mg/kg (group 5) immediately prior to each exposure.

**Results:**

No tumours were seen in the control group. The animals in group 2 (no amifostine) developed squamous cell carcinoma (SCC) at 3.5–4.5 months (mean 3.9 months). Groups 3 and 4 (low- and medium-dose amifostine) developed SCC at 4.0–7.0 months (mean 5.3 months), representing a statistically significant delay in tumour presentation (p = 0.04). An even greater delay was seen in group 5 (high-dose amifostine), which developed SCC at 7.0–9.0 months (mean 8.5 months, p < 0.001 versus groups 3 and 4). Ocular keratitis developed in all animals except the unexposed controls and the high-dose treatment group.

**Conclusion:**

Treatment with amifostine significantly delays the onset of skin cancer and prevents ocular keratitis in UVB-exposed XP mice.

## Introduction

Skin cancer is the most common malignancy in the United States, affecting over one million patients annually and representing one-third of all cancer diagnoses [1]. Exposure to solar ultraviolet radiation, particularly ultraviolet B (UVB), is the predominant cause of most skin cancers acting through mechanisms of DNA damage (either directly or via free-radical generation) and immune system inhibition [2, 3]. The development of skin cancer is also linked to several patient characteristics, including fair skin, light hair and eye colour, the tendency to burn rather than tan, and family history [2].

The combination of individual susceptibility and environmental exposure is particularly devastating to patients with xeroderma pigmentosum (XP), a disorder characterized by an inability to repair damaged DNA. Patients with this disorder have a 1000-fold increased incidence—and essentially a 100% lifetime incidence—of skin cancer, usually presenting at a young age [4]. Standard measures for skin cancer prevention, such as sun avoidance, protective clothing, and sunscreens, are inadequate in XP patients [5], underscoring the need for more effective prophylaxis.

Amifostine (Ethyol; MedImmune, Inc., Gaithersburg, MD), is an aminothiol compound known for its cytoprotective effects. Its active metabolite is an intracellular free radical scavenger that prevents interstrand DNA crosslinking. Originally developed by the military during the Cold War to protect personnel from radiation sickness, it has since been used clinically to ameliorate the side effects of chemotherapy and radiotherapy [6]. Given its ability to scavenge free radicals and to protect DNA architecture, we hypothesized that amifostine could inhibit the development of skin cancer in XP mice exposed to UVB.

## Materials and methods

This protocol was approved by the Animal Care and Use Committee (ACUC) of the University of Missouri-Columbia.

Twenty-five XP mice aged 7–8 weeks were randomly divided into five equal groups (Table 1). The animals in group 1 served as controls, receiving no UVB exposure and no treatment with amifostine. The animals in group 2 were exposed to UVB every other day at a dose of 200 mJ/cm^2^ (10 mJ/cm^2^/min for 20 minutes), using a Panasol II UVB lamp (National Biological Corp., Twinsling, OH). They received intraperitoneal injections of saline prior to each exposure, and thus served as a placebo group. The remaining three groups received the same irradiation, but were treated with intraperitoneal injections of amifostine prior to each exposure. In group 3, the animals received amifostine at a low dose of 50 mg/kg; in group 4, a medium dose of 100 mg/kg; and in group 5, a high dose of 200 mg/kg. The high dose was well below the reported LD50 for amifostine in mice (550–1140 mg/kg) [6].

The animals were housed in our institution’s animal care facility according to ACUC guidelines and had minimal additional ultraviolet exposure. They were examined every other day, at the time of their injections and irradiation, and suspicious lesions were biopsied. The study was continued until all non-control animals had developed skin cancer, which occurred at nine months. At this time all animals were euthanized and additional lesions were biopsied.

Statistical calculations were performed with one-way analysis of variance using the Holm–Sidak method of pair-wise multiple comparison procedures.

## Results

Three animals died during the course of the study. One animal from group 4 (medium-dose amifostine) and one from group 5 (high-dose amifostine) died at approximately two months as a result of intrasplenic injection. Postmortem examination revealed no evidence of malignancy in either animal; however, they were excluded from statistical analysis because the remaining subjects in their respective groups developed tumours in a significantly later timeframe. The third animal, from group 2 (placebo), died as a result of complications from metastatic squamous cell carcinoma (SCC) after four months of UVB exposure.

**Figure 1:**
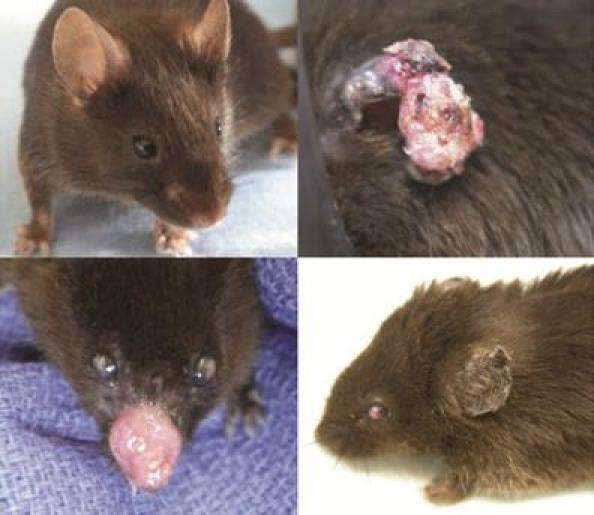
No tumours developed in non-exposed control mice (*above, left*) throughout the nine-month study period. Squamous cell carcinoma developed in all ultraviolet B-exposed mice, most commonly on the ears (*above, right*) and nose (*below, left*). All ultraviolet B-exposed mice developed ocular keratitis (*below, left and right*), with the exception of those receiving high-dose amifostine.

No tumours were seen in any of the control animals (group 1). All of the UVB-exposed animals, with the exception of the two that died early from intrasplenic injections, developed SCC (Fig. 1). No other types of skin cancer (e.g., basal cell carcinoma or melanoma) were identified.

SCC was first seen in group 2 (placebo) at 3.5 months, with all animals affected by 4.5 months (mean 3.9 months).

SCC appeared in group 3 (low-dose amifostine) at 4.0–7.0 months (mean 5.5 months). The surviving animals in group 4 (medium-dose amifostine) developed SCC in a similar timeframe (4.0–7.0 months, mean 5.1 months). This onset represents a statistically significant delay in tumour presentation compared to group 2 (placebo) (p = 0.04).

An even greater delay was seen in the surviving members of group 5 (high-dose amifostine), which developed SCC at 7.0–9.0 months (mean 8.5 months). When compared to the onset in groups 3 and 4, this delay was highly statistically significant (p < 0.001) (Table 1).

In addition to SCC, ocular keratitis developed in all animals except those in group 1 (non-exposed controls) and group 5 (high-dose amifostine) (Fig. 1). Despite the keratitis, no ocular tumours were identified in any of the animals.

## Discussion

This study demonstrated the chemopreventive capacity of amifostine in the setting of UVB exposure.

Chemoprevention is the use of chemical agents to prevent the development of cancer [7]. As summarized by Harris and Alberts [8], the ideal chemopreventive agent displays minimal toxicity in healthy tissues and can differentiate cancerous from healthy cells. In addition, it should be tailored to act within the carcinogenic cascade of a particular malignancy in order to have a “rational mechanism of action”. In designing this study, amifostine was thought to fulfil these criteria and to be an appropriate chemopreventive agent in the development of UVB-induced skin cancer.

Amifostine was developed by the Walter Reed Army Institute of Research in the 1970s as part of a classified project investigating agents that could be used to protect military personnel in the event of a nuclear war [9]. Amifostine had the greatest radioprotective capability and the best safety profile of 4,400 compounds evaluated at that time [6]. Its active metabolite is generated by the interaction of the parent drug with membrane-bound alkaline phosphatase. Because this enzyme is usually present only in normal tissues, the active metabolite is able to enter only healthy cells, where it exerts at least two cytoprotective actions: scavenging of free radicals (generated chemically or by ionizing radiation) [10] and prevention of interstrand DNA crosslinking [11].

Either or both of these actions may be responsible for the cytoprotective effect of amifostine on keratinocytes. Like all other cancers, SCC follows a step-wise progression from normally functioning cells to malignant neoplasm. In the case of SCC, the transformation begins with actinic keratosis, which transitions to carcinoma in situ, and, finally, to SCC [12]. The instigating factors are variable and can involve free radical damage, pyrimidine dimer formation, DNA repair defects, and mutations in tumour suppressor genes [13, 14]. Each step of this progression is necessary for tumour formation, and each step could theoretically be blocked by the action of amifostine.

The XP mouse is at significantly increased risk for the development of skin cancer (particularly SCC) due to its inability to repair damaged DNA [4]. Because disease progression was significantly delayed, it is probable that amifostine functioned as a DNA protectant as described above, but was overwhelmed by the UVB effect in this highly susceptible population. A similar pattern was evident with regard to ocular keratitis, a condition seen in about 40% of XP patients [15], but in none of the animals in the high-dose treatment group. This observation further supports the presumption that amifostine at the studied doses inhibited DNA damage in this model, but was not completely adequate in cutaneous tissues.

Sunscreens and photoprotective clothing are the current mainstays in the prevention of solar radiation-induced skin cancer. While both modalities can be protective, compliance is often impractical and sporadic, limiting their clinical effectiveness [16–18]. Various dietary interventions have also been recommended as a means of preventing skin cancer [19], but in most cases (including supplementation with selenium, beta-carotene, and vitamin E), scientific trials have failed to demonstrate a chemopreventive potential [20–22]. Notable exceptions include dietary supplementation with retinoids and restriction of dietary fat, which have both been shown to produce a modest reduction in the incidence of skin cancer [23, 24]. Nonetheless, currently available prophylaxis against skin cancer is greatly limited, making effective chemoprevention an attractive addition to the clinical armamentarium.

Amifostine is currently approved for the reduction of cisplatin-induced nephrotoxicity in patients being treated for ovarian or lung cancer, and radiation-induced xerostomia in patients being treated for head and neck cancer. It is administered as an intravenous infusion shortly before chemotherapy or radiotherapy. The recommended starting dose is 910 mg/m^2^ (approximately 25 mg/kg in the average adult), with a maximum dose of 1300 mg/m^2^ (approximately 35 mg/kg). The most common adverse reactions are transient hypotension and nausea/vomiting [25]. Although the doses used in this study were well below the LD50 for mice, they were substantially above the recommended doses for humans [6]. This fact represents an obvious hurdle in the clinical application of amifostine as a chemopreventive option for humans, as does the current availability of amifostine only as an intravenous infusion. Nonetheless, amifostine appears to be an efficacious and well-tolerated chemopreventive agent in XP mice, and with further study and development may become a feasible option for humans, as well.

A limitation of this study is its rather small number of subjects. Although preliminary analysis indicated a sufficient quantity in each group, the loss of two subjects from iatrogenic injury may have compromised the power of the study. Thus, while our results were both statistically significant and logically consistent, further and more extensive investigations are clearly warranted.

## Figures and Tables

**Table 1:**
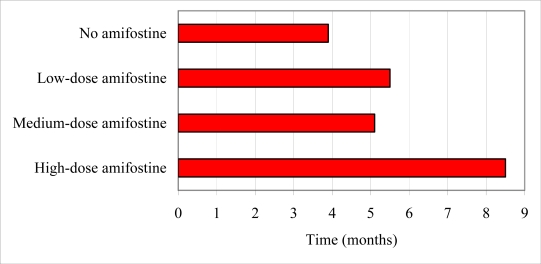
Mean time to tumour development in ultraviolet B-exposed xeroderma pigmentosum mice. Error bars indicate the range of onset. No tumours developed in non-exposed controls.
